# Atrial fibrillation ablation using three-dimensional artificial intelligence module integration with intracardiac echocardiography

**DOI:** 10.1093/europace/euag009

**Published:** 2026-01-16

**Authors:** Fengwei Zou, Sarah Xu, Sanjana Nagraj, Sheetal Mathai, Ariel Gidon, Nay Yee Wint Kyaw, Jose Matias, Giuseppe Ammirati, Jacopo Marazzato, Aung Lin, Domingo Y Ynoa, Marco Schiavone, Vincenzo Mirco La Fazia, Sanghamitra Mohanty, Andrea Natale, Pasquale Santangeli, Xiaodong Zhang, Luigi Di Biase

**Affiliations:** Montefiore Einstein Center for Heart & Vascular Care, Montefiore Medical Center, Albert Einstein College of Medicine, 111 E 210th Street, Bronx, NY 10467, USA; Montefiore Einstein Center for Heart & Vascular Care, Montefiore Medical Center, Albert Einstein College of Medicine, 111 E 210th Street, Bronx, NY 10467, USA; Montefiore Einstein Center for Heart & Vascular Care, Montefiore Medical Center, Albert Einstein College of Medicine, 111 E 210th Street, Bronx, NY 10467, USA; Montefiore Einstein Center for Heart & Vascular Care, Montefiore Medical Center, Albert Einstein College of Medicine, 111 E 210th Street, Bronx, NY 10467, USA; Montefiore Einstein Center for Heart & Vascular Care, Montefiore Medical Center, Albert Einstein College of Medicine, 111 E 210th Street, Bronx, NY 10467, USA; Montefiore Einstein Center for Heart & Vascular Care, Montefiore Medical Center, Albert Einstein College of Medicine, 111 E 210th Street, Bronx, NY 10467, USA; Montefiore Einstein Center for Heart & Vascular Care, Montefiore Medical Center, Albert Einstein College of Medicine, 111 E 210th Street, Bronx, NY 10467, USA; Montefiore Einstein Center for Heart & Vascular Care, Montefiore Medical Center, Albert Einstein College of Medicine, 111 E 210th Street, Bronx, NY 10467, USA; Montefiore Einstein Center for Heart & Vascular Care, Montefiore Medical Center, Albert Einstein College of Medicine, 111 E 210th Street, Bronx, NY 10467, USA; Montefiore Einstein Center for Heart & Vascular Care, Montefiore Medical Center, Albert Einstein College of Medicine, 111 E 210th Street, Bronx, NY 10467, USA; Montefiore Einstein Center for Heart & Vascular Care, Montefiore Medical Center, Albert Einstein College of Medicine, 111 E 210th Street, Bronx, NY 10467, USA; Centro Cardiologico Monzino, IRCCS, Milan 20138, Italy; Texas Cardiac Arrhythmias Institute, St. David’s Medical Center, Austin, TX 78705, USA; Texas Cardiac Arrhythmias Institute, St. David’s Medical Center, Austin, TX 78705, USA; Texas Cardiac Arrhythmias Institute, St. David’s Medical Center, Austin, TX 78705, USA; Cardiac Electrophysiology and Pacing, Department of Cardiovascular Medicine, Cleveland Clinic, Cleveland, OH 44195, USA; Montefiore Einstein Center for Heart & Vascular Care, Montefiore Medical Center, Albert Einstein College of Medicine, 111 E 210th Street, Bronx, NY 10467, USA; Montefiore Einstein Center for Heart & Vascular Care, Montefiore Medical Center, Albert Einstein College of Medicine, 111 E 210th Street, Bronx, NY 10467, USA

**Keywords:** Atrial fibrillation, Catheter ablation, Electroanatomical mapping, Intracardiac echocardiography, CARTOSOUND FAM, Artificial intelligence

## Abstract

**Aims:**

Intracardiac echocardiography-based electroanatomical mapping (EAM) improves procedural efficiency and safety in atrial fibrillation (AF) ablation and remains the standard of care. The CARTOSOUND FAM (AI FAM) module uses a deep-learning algorithm that automates left atrial reconstruction without manual contouring. This study aims to evaluate the 1-year outcomes of AI FAM compared to standard-of-care EAM in AF ablation.

**Methods and results:**

This study included 298 patients undergoing radiofrequency AF ablation between January 2021 and December 2023. Patients treated before January 2023 underwent standard-of-care EAM, while those in 2023 utilized AI FAM-based reconstruction. Baseline demographics, comorbidities, AF type, and medication use were recorded. Procedural characteristics, acute success, complications, and AF recurrence at 1-year follow-up were analysed. Of the 298 patients, 115 underwent mapping with AI FAM and 183 with EAM. Baseline characteristics were comparable. AI FAM reduced mean total procedure time (122.5 ± 23.5 vs. 129.0 ± 30.4 min, *P* = 0.040) and left atrial (LA) dwell time (78.3 ± 21.45 vs. 87.5 ± 28.2 min, *P* = 0.001). Acute procedural success was 98.3% in AI FAM vs. 98.9% in EAM with fewer complications observed in the AI FAM group (1 vs. 4). At 1 year, freedom from AF recurrence was comparable (80.0% AI FAM vs. 81.4% EAM at 1 year, LogRank *P* = 0.610).

**Conclusion:**

AI FAM was associated with incremental but significant procedural advantages over conventional contouring via reduced total procedure time and LA dwell time, without compromising acute and long-term safety and rhythm control efficacy. AI FAM integration with pulsed field ablation will mark another step towards making AF ablation more streamlined and accessible.

## Graphical Abstract

**Graphical Abstract:**
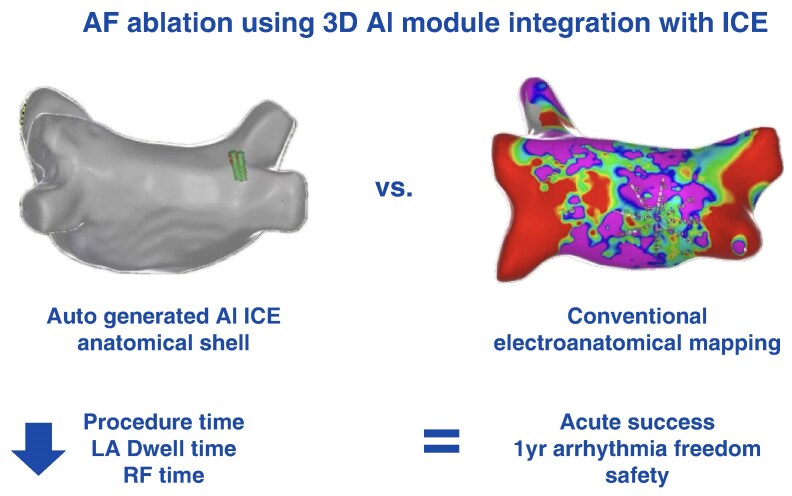

What's new?

- AI-ICE mapping automates left atrial reconstruction, reducing need for manual contouring.
- In routine RF AF ablation, this automation improves procedural efficiency without sacrificing safety and long term efficacy.
- One-year rhythm outcomes are comparable to standard electroanatomical mapping.
- AI-ICE mapping supports more streamlined AF ablation workflows in the modern era.

## Introduction

The widespread utilization of intracardiac echocardiography (ICE) represents a major advancement in cardiac imaging for interventional cardiology and electrophysiology procedures.^1,2^ Intracardiac echocardiography allows for real-time assessment of relevant cardiac structures with catheter manipulation, enabling early recognition of cardiac complications while limiting radiation exposure.^3^ The CARTO^TM^ 3 System (Biosense Webster, Irvine, CA) is a three-dimensional (3D) electroanatomical mapping (EAM) system using magnetic sensors and a low magnetic field generating pad to achieve high spatial resolution in creating 3D maps.^4^ In its 8th iteration (version 8), the CARTOSOUND^TM^ FAM (AI FAM) module integrates a deep-learning artificial intelligence (AI) algorithm that automates the process of 3D left atrial (LA) anatomical shell reconstruction via rotations of the ICE crystal without the need for dedicated mapping catheters to perform EAM.

The feasibility of the AI FAM module has been previously studied, showing high fidelity in identifying LA anatomical structures such as the pulmonary veins (PVs) and left atrial appendage (LAA). AI FAM demonstrated comparable ostial diameters, carina-to-carina distances, and LA image-based measurements and qualitative evaluation compared to pre-procedural CT with highly reliable inter-operator reproducibility.^5^ Other proposed advantages of the AI FAM module include decreased total procedure time due to the elimination of mapping time and potentially reducing adverse events by decreasing the number of sheath exchanges. Our earlier experience incorporating the AI FAM module into daily clinical practice witnessed excellent acute success rate and safety profile.^6^ This study aims to evaluate the long-term arrhythmia-free outcomes of routine AI FAM practice compared to standard-of-care EAM in patients undergoing AF ablation.

## Methods

**Figure 1:**
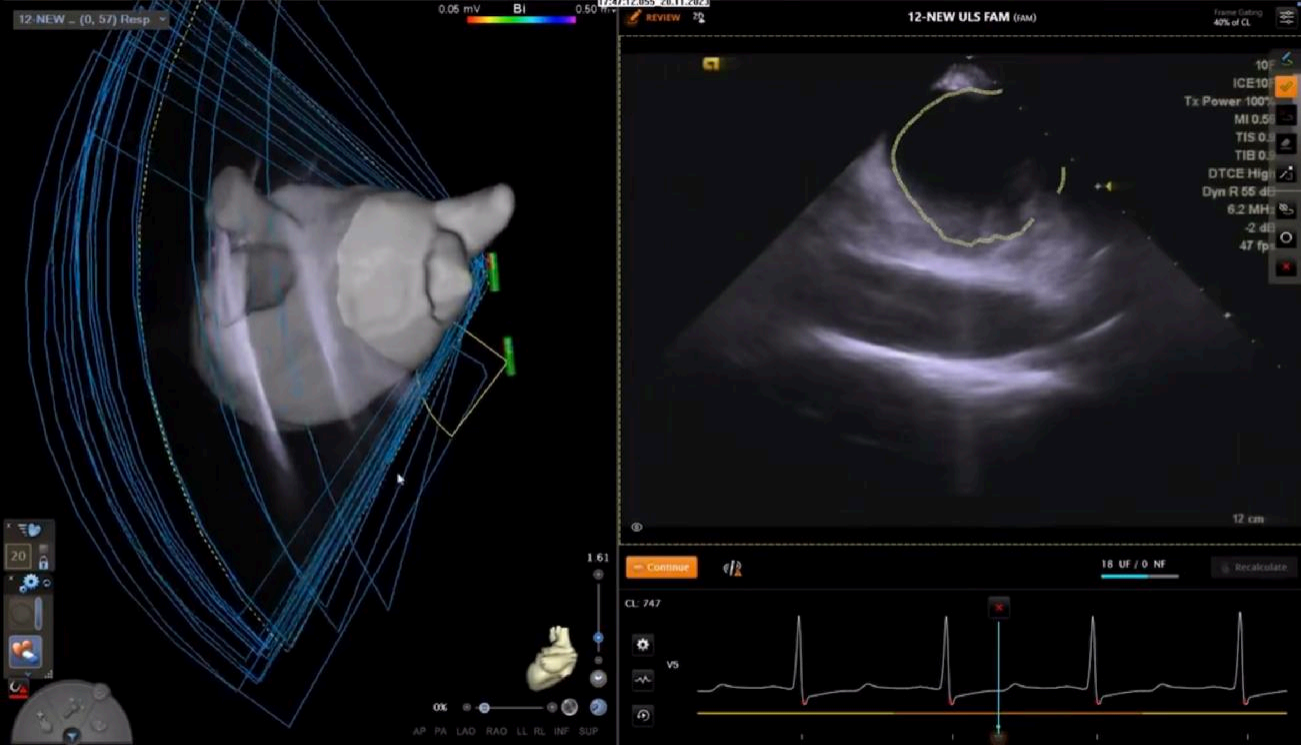
AI FAM automatic anatomical acquisition. The LA shell was automatically reconstructed by rotating the ICE crystal to cover the whole anatomy of the LA body, PVs, and the LAA (left panel, Supplementary material online, *Video S1*). The AI module identified anatomical borders by creating corresponding tags (right panel, encircled lines).

Patients undergoing AF radiofrequency (RF) ablation in a high-volume US centre from January 2021 to December 2023 were retrospectively included in this study. All patients received general anaesthesia for the procedure. After vascular access was obtained, an ICE catheter was used to visualize the right atrium, tricuspid valve, fossa ovale, coronary sinus, overall LA, aortic valve, LAA, left superior PV, left inferior PV, right superior PV, and right inferior PV. In patients undergoing AF ablation before January 2023, ICE was used only to identify anatomical structures, guide transseptal puncture, and monitor catheter contact as well as complications. In those patients, after achieving transseptal access, conventional EAM was performed using a dedicated mapping catheter to create the anatomical shell of the LA and essential landmarks as mentioned above (EAM group). In patients undergoing ablation after January 2023, the AI FAM module was used to automatically reconstruct the LA anatomy without a mapping catheter (AI FAM group) before the initial energy delivery. In this group, mapping catheter use is allowed for remapping after PVI and/or to study and target non-PV triggers. Descriptions of the AI FAM algorithm as well as best practice recommended workflow to accurately and efficiently create the LA anatomy were also previously summarized.^5,7^ As previously reported, the average calculation time for LA anatomical reconstruction was 65 s.^5^ A graphic and video illustration of the AI FAM programme is shown in *Figure 1* and Supplementary material online, *Video S1*. Radiofrequency ablations were carried out using the THERMACOOL SMARTTOUCH SF (STSF) catheter (Biosense Webster, Irvine, CA). As previously reported, ablation parameters included an RF power range of 15–45 W with a contact force range of 5–25. The VISITAG stability recommended settings were 2–3 mm across 3–5 s, and the recommended tag size was 3 mm with an inter-tag distance of ≤6 mm, targeting the VISITAG SURPOINT ablation index of 550 for the anterior, ridge, and roof segments and 400 for the posterior and inferior segments.^8^ The target values could be lowered at the operator’s discretion to address safety concerns. All patients proceeded with ablation without changes to routine institutional procedure workflow or limitations to the lesion sets deemed necessary by the primary operator.

Baseline demographic information as well as comorbidities and medication use were recorded. Procedure-related information including total procedure time (skin-to-skin time), fluoroscopy time, RF energy application time, LA dwell time, AF ablation lesion sets, complications, and acute success were summarized. Acute success is defined as conduction block in/out of each PV for PV isolation and absence of any electrical potentials in ablation of non-PV triggers at the end of ablation.

All patients received routine follow-up, and the follow-up protocol was identical between the two groups. Routine clinical follow-up was performed at 1 month, 6 months, 12 months, and yearly after the procedure. Fourteen-day continuous rhythm monitors were prescribed at 5 months and 11 months and were reviewed at the following follow-up visit. ECGs were performed in all clinic visits, and patients were encouraged to contact the providers should symptoms occurred to expedite rhythm confirmation.

Freedom from atrial fibrillation recurrence (AF, atrial tachycardia, or atrial flutter exceeding 30 seconds) was evaluated with or without the use of antiarrhythmic medications (AADs). Atrial arrhythmia recurrence occurring in the first 90 days after the ablation was considered to be within the blanking period. This study is approved by the institutional review board.

## Statistical analysis

Continuous variables were calculated as means ± SD and compared using independent *t*-tests. Categorical variables were presented as frequency (%) of the total cohort and were compared using χ^2^ tests. Freedom from atrial arrhythmia recurrence over time was analysed using Kaplan–Meier survival curves and utilizing the log-rank test. Restricted mean survival time (RMST) was also calculated as area under the Kaplan–Meier survival curve and compared to assess recurrence-free survival. A two-sided *P* value of <0.05 was considered statistically significant.

## Results

### Study population

A total of 298 patients were included in this study with 115 undergoing mapping with AI FAM and 183 with EAM. The baseline demographic information, comorbidities, and medication use were summarized in *Table 1*. The average age was 65.7 ± 9.5 years old with 33% female in the AI FAM group compared to 66.4 ± 11.4 years old with 39% female in the EAM group. Both groups had similar BMI. Comorbidities including CAD, Type 2 diabetes mellitus (T2DM), hypertension (HTN), congestive heart failure (CHF), chronic kidney disease (CKD)/ESRD, transient ischaemic attack (TIA)/stroke, and obstructive sleep apnoea (OSA) were all comparable between groups without statistical significance.

**Table 1:** Baseline characteristics

	AI FAM (*n* = 115)	EAM (*n* = 183)	*P* value
Female	38 (33.0%)	72 (39.3%)	0.273
Age	65.7 ± 9.5	66.4 ± 11.4	0.619
BMI	31.2 ± 6.9	31.2 ± 7.4	0.985
CAD	37 (32.2%)	48 (26.2%)	0.408
T2DM	34 (29.6%)	63 (34.4%)	0.383
HTN	93 (80.9%)	148 (80.9%)	0.999
HLD	50 (43.5%)	113 (61.7%)	0.002
CHF	40 (35.1%)	54 (29.5%)	0.315
LVEF (%)	55.3 ± 11.8	55.9 ± 13.3	0.69
LA diameter (cm)	4.3 ± 0.8	4.3 ± 0.8	0.741
CKD/ESRD	20 (17.4%)	20 (10.9%)	0.111
COPD	11 (9.6%)	6 (3.3%)	0.023
TIA/stroke	15 (13.0%)	29 (15.8%)	0.507
OSA	18 (15.7%)	38 (20.8%)	0.271
CHA_2_DS_2_-VASc	3.0 ± 1.8	3.1 ± 1.7	0.487
Beta-blocker	87 (75.7%)	146 (79.8%)	0.401
Calcium channel blocker	22 (19.1%)	25 (13.7%)	0.207
Oral anticoagulation			0.450
Apixaban	77.2%	75.4%	
Rivaroxaban	18.4%	16.4%	
Dabigatran	0.0%	1.6%	
Warfarin	4.4%	6.6%	
AF type			0.853
Paroxysmal	59 (51.3%)	88 (48.1%)	
Persistent	50 (43.5%)	84 (45.9%)	
LSPersistent	6 (5.2%)	11 (6.0%)	
Prior AF ablation	33 (28.7%)	50 (27.3%)	0.797

The only two comorbid conditions of statistical difference were hyperlipidaemia (43.5% AI FAM vs. 61.7% EAM) and chronic obstructive pulmonary disease (9.6% AI FAM vs. 3.3% EAM). The mean LA diameter was 4.3 cm in both groups, and left ventricular ejection fraction (LVEF) was 55.3 ± 11.8% in AI FAM vs. 55.9 ± 13.3% in EAM. Similar use of beta-blockers, calcium channel blockers, oral anticoagulation, patients with previous AF ablations, and AF-type distribution (paroxysmal vs. persistent vs. long-standing persistent) were also observed without significant in-group difference.

### Ablation characteristics

Ablation characteristics for both groups are presented in *Table 2*. The majority of patients underwent pulmonary vein isolation (PVI) ablation (92.2% AI FAM vs. 90.2% EAM), with exceptions being redo AF ablation when all PVs were confirmed isolated from previous ablations. Other lesion sets including posterior wall isolation (PWI), coronary sinus isolation (CS), and cavotricuspid isthmus ablation (CTI) were similar. Patients in the AI FAM group received more superior vena cava (SVC) isolation (74.8% vs. 46.4%, *P* < 0.001). The total skin-to-skin procedure time in the AI FAM group was 122.5 ± 23.5 min, which was shorter than 129.0 ± 30.4 min in the EAM group while reaching statistical significance (*P* = 0.046). The mean RF energy application time was also shorter in the AI FAM group (28.0 ± 9.8 vs. 31.8 ± 12.5 min). The mean LA dwell time was also shorter in the AI FAM group compared to the EAM group by 9.2 min (78.3 ± 21.4 vs. 87.5 ± 28.2 min, *P* = 0.001). Acute success was achieved over 98% in both groups. The only complication in the AI FAM group was a right femoral arteriovenous fistula, while a total of four complications occurred in the EAM group (one pericardial effusion requiring drainage, one LA perforation requiring surgical repair, one CVA, and one sinus node dysfunction requiring permanent pacemaker implantation for concurrent antiarrhythmic use).

**Table 2:** Ablation characteristics

	AI FAM (n = 115)	EAM (n = 183)	*P* value
Skin-to-skin procedure Time (min)	122.5 ± 23.5	129.0 ± 30.4	0.040
LA dwell time (min)	78.3 ± 21.4	87.5 ± 28.2	0.001
RF energy time (min)	28.0 ± 9.8	31.8 ± 12.5	0.006
AF ablation lesion set			
PVI	106 (92.2%)	165 (90.2%)	0.712
PWI	72 (62.6%)	131 (71.6%)	0.106
SVC	86 (74.8%)	85 (46.4%)	<0.001
CS	27 (23.5%)	46 (25.1%)	0.746
CTI	16 (13.9%)	27 (14.8%)	0.841
Acute success	113 (98.3%)	181 (98.9%)	0.637
Complications	1 (0.9%)	4 (2.2%)	0.389

### Rhythm control efficacy

**Figure 2:**
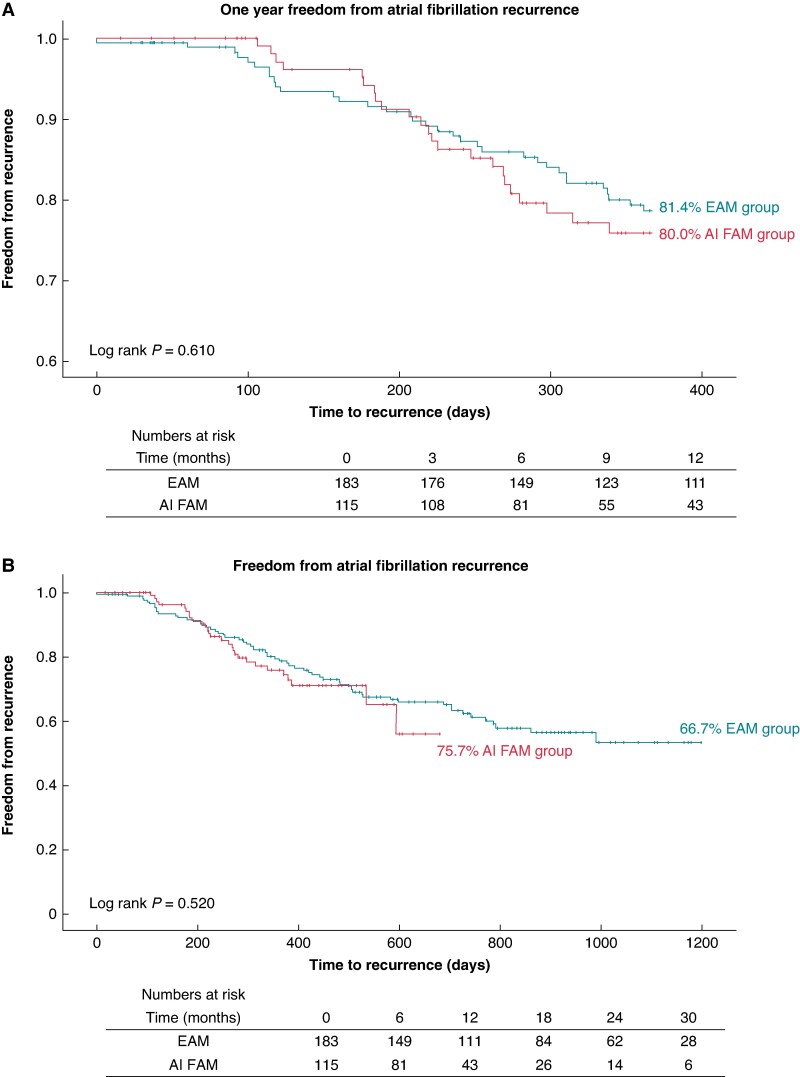
Freedom from atrial fibrillation recurrence at 1 year and maximum follow-up.

**Figure 3:**
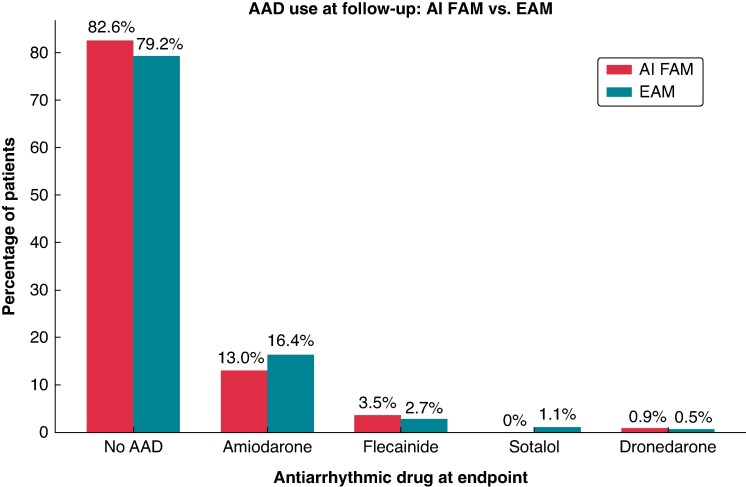
Antiarrhythmic medication use.

At 1-year follow-up, freedom of AF recurrence was achieved in 80.0% in the AI FAM group compared to 81.4% in the EAM group without a statistical difference (LogRank *P* = 0.610) as demonstrated in the Kaplan–Meier curve in *Figure 2A*. The overall mean follow-up time was 455.7 days for the entire cohort with 330.2 days for AI FAM and 534.5 days for EAM. At maximum follow-up duration, freedom of AF recurrence was achieved in 75.7% in the AI FAM group compared to 66.7% in the EAM group without statistical difference (LogRank *P* = 0.520, *Figure 2B*). The majority of patients were not taking AADs at the time of follow-up or recurrence as shown in *Figure 3*. The 1-year freedom from AF recurrence off AAD was 83.5% in the EAM group vs. 76.6% in the AI FAM without statistical significance (*P* = 0.156). The most common AADs in descending orders of use were amiodarone, flecainide, sotalol, and dronedarone with comparable use across the two groups. The restricted mean survival time for the AI FAM group was 330.4 days compared to 332.1 days in the EAM group (*P* = 0.85) in all patients and 333.6 days vs. 337.8 days in patients off AAD (*P* = 0.66).

## Discussion

The main findings of this study were as follows: (i) AI-based FAM module was associated with reduced AF ablation procedure time and LA dwell time compared to conventional EAM by eliminating the need for pre-ablation contact mapping; (ii) AI FAM-assisted AF ablation can be safely performed routinely without changing ablation workflow; and (iii) using AI-based FAM module to generate LA geometry without EAM is effective in achieving both acute and long-term success without compromising rhythm control efficacy compared to standard-of-care EAM. To the best of our knowledge, this is the first study directly comparing acute success and long-term arrhythmia freedom in patients receiving AF ablation with the AI FAM alone without pre-ablation contact mapping vs. conventional EAM.

With increasing evidence over the past decade, catheter ablation is gaining popularity as the first-line rhythm control strategy for patients with AF.^9,10^ In the 2023 ACC/AHA/ACCP/HRS Guideline for the Diagnosis and Management of Atrial Fibrillation, AF ablation received a Class I recommendation for symptomatic AF patients desiring rhythm control.^10^ Recent advancements in ablation technology including pulsed-field energy source have made ablation more accessible to the increasing number of AF patients.^11,12^ In an effort to further improve the efficiency of AF ablation, the deep-learning-integrated CARTOSOUND^TM^ FAM module builds on the existing CARTO EAM system. It is intended to simplify the workflow of anatomical reconstruction using automated tagging of ultrasound images obtained from ICE and eliminate the need for manual EAM with dedicated contact mapping catheters. As AI is increasingly integrated with the field of electrophysiology, rigorous description and testing of its function is necessary.^13^ Early clinical studies evaluated the accuracy of reconstructed LA anatomies such as ostial diameters of PVs and LAA as well as carina-to-carina distance against pre-ablation cardiac computed tomography (CT) images and demonstrated good accuracy and interoperative variability.^5^ Direct comparison of AI FAM with contact-based EAM in patients undergoing ablation also showed that the median distance of the ablation catheter was <5 mm from the AI FAM reconstruction in all PVs. Important anatomical landmarks such as fossa ovale for transseptal puncture were reproduced with high fidelity to guide critical steps in ablation.^14^

So far, most evidence accumulated for AI FAM pertained to its feasibility to be incorporated into standard ablation workflows and rarely reported its impact on rhythm control efficacy. One potential concern for the widespread adaptation of AI-based FAM is whether adequate tissue-catheter contact can be achieved based on the anatomical shell created with AI FAM alone. Tissue-catheter contact has repeatedly been demonstrated to be one of the most critical components of durable lesion formation, and its integration into the ablation index was shown to improve the rate of first-pass isolation and freedom from AF recurrence.^15^ Previous experience from our centre has demonstrated that acute ablation success can be achieved in 98% of patients undergoing AF ablation solely using the AI FAM module.^6^ In this cohort, no immediate complications were observed. Despite promising results, the lack of a direct comparison group and long-term arrhythmia recurrence follow-up limited the interpretation of results. In this current study, patients receiving AF ablation with AI FAM had similar baseline comorbidities, CHA_2_DS_2_-VASc score, medication use, AF-type distribution, and key echocardiographic parameters such as LVEF and LA dimension compared to their counterparts in the conventional EAM ablation groups. These indicated both groups have very similar AF substrates to allow for reliable interpretation of efficacy results. In terms of ablation targets, the majority of patients in both groups received PVI (>90%) and PWI (60–70%). Other non-PV triggers such as CS and CTI were also similarly targeted. We did observe that more SVC ablations were performed in the AI FAM group. However, SVC potentials were thoroughly assessed prior to ablation, and no ablation was performed if SVC lacked electrical signals indicating similar functional end results to achieve lack of electrical potential in the SVC at the end of ablation for both groups.

In terms of procedure time, the total skin-to-skin time is shorter in the AI FAM group by 6.5 min and reached statistical significance. While RF energy time was slightly shorter in the AI FAM group by 3.5 min, the LA dwell time was shorter by 9.2 min. Since the time to generate the AI FAM map was around 60 s, we believe the additional decrease in LA dwell time was most likely attributed to the reduced number of catheter changes and lack of contact mapping, which ultimately contributed to shorter overall skin-to-skin procedure time (∼6 min).

At both 1 year and maximum follow-up, patients in both groups had similar rates of AF recurrence both with and without AAD use. At follow-up, the majority of patients in both groups were not taking AADs (82.6% vs. 79.2%), and the distribution of AADs used was also similar (*Figure 3*). The restricted mean survival time analysis for both groups further confirmed that RF ablation using the LA shell generated by AI FAM did not compromise long-term freedom from AF recurrence with or without AAD. These results indicate accuracy of the initial anatomical reconstruction by AI FAM and lesion durability that is comparable to EAM.

In this study, we observed lower complication rates in the AI FAM group compared to conventional EAM (0.9% vs. 2.2%). We believe AI FAM integration into standard ablation workflow has the potential to decrease procedural-related complications mainly due to the reduced number of catheter exchanges and contact mapping. The only complication from the AI FAM group was a vascular complication that is unrelated to the AI FAM technology studied. Conversely, in the conventional EAM group, one stroke was observed and was likely related to air introduction during sheath exchange. Thromboembolism is a well-known complication associated with catheter ablations, particularly RF ablations. The red blood cell destruction and platelet release during RF create a prothrombotic environment with activated cell membranes and the release of clotting factors.^16^ Previous studies suggest that catheter manipulation, such as catheter sheath introduction and exchanges, impacts thrombogenicity significantly and that longer procedure durations are associated with increased catheters inserted and have more energy applications.^17^ By eliminating the need for mapping catheters during initial anatomical reconstruction, AI FAM-integrated ablation at least reduces one catheter/sheath exchange prior to energy delivery, which might explain the decreased risk of complications in this cohort. Since this study is not powered for safety endpoints, the catheter exchange frequency using AI FAM and its impact on stroke incidence is an area that should be investigated further.

In this current pulsed-field ablation era, AI FAM was also demonstrated to seamlessly integrate with the variable loop circular mapping and electroporation ablation catheter using the TRUPULSE™ Generator from Biosense Webster. In a single-centre series of 11 patients, the total skin-to-skin pulsed field ablation (PFA) ablation time was reduced to 50 min for PVI alone using the above-mentioned workflow.^18^ From our own experience integrating AI FAM and PFA, in many patients undergoing index PVI with or without extra-PV trigger ablation, AI FAM was able to improve workflow. An ongoing study from our institution on the safety and efficacy of AI FAM integration with variable loop circular catheter PFA system is also underway. Moreover, in a recent preclinical study, CARTOSOUND FAM integration with 4D ICE was investigated in swine models on ablating ventricular structures such as RV moderator band and LV papillary muscles as well as analysing LAA anatomy guided only by ICE images. Autopsy results showed a good correlation with ICE images, EAM, and pathological examination.^19^ These early results opened doors for more potential utilization of AI-based FAM modules in procedures such as ventricular arrhythmia ablation and concomitant AF ablation and LAA occlusion.

## Limitations

This study has several limitations. First, this was a non-randomized single-centre experience that may limit the generalizability of the study results. While this was a retrospective chronological cohort, we have included cases from the same operators to minimize inter-operator variability that could impact mapping and ablation time, and the same mapping catheters, ablation catheters, and ablation approaches have been used during the time of this study. Second, as a retrospective cohort study, there was limited control in the variability of baseline characteristics and follow-up, which intrinsically weakens the strength of the results. Additionally, the current deep-learning algorithm is limited in processing images with unusual anatomical variations, such as a fifth PV. The programme may need further training to accommodate such anatomical variations, which may limit its generalizability. Last but not least, the AI FAM module does not generate voltage maps and will require additional mapping catheters to identify areas of electrical activity when non-PV triggers are targeted after initial PVI.

## Conclusion

AI FAM was associated with incremental but significant procedural advantages over conventional contouring via reduced total procedure time, RF time, and LA dwell time, without compromising acute and long-term safety and success to achieve rhythm control. AI FAM integration with PFA will mark another step towards making AF ablation more streamlined and accessible.

## Supplementary Material

euag009_Supplementary_Data

## Data Availability

The data underlying this article will be shared on reasonable request to the corresponding author.
